# Topological regions and experimental carcinogenicity data of polycyclic aromatic hydrocarbons: a comprehensive resource for prediction models

**DOI:** 10.1186/s13104-026-07791-w

**Published:** 2026-04-03

**Authors:** Meressa Welearegay, Andrzej Holas, Robert Balawender, Paul W. Ayers, Matthew Chan, Farnaz Heidar-Zadeh

**Affiliations:** 1 Department of Chemistry, Mekelle University, Mekelle, Ethiopia; 2 Institute of Physical Chemistry, Polish Academy of Sciences, Warsaw, Poland; 3 Department of Chemistry and Chemical Biology, McMaster University, Hamilton, Canada; 4 Department of Chemistry, Queen’s University, Kingston, Canada

**Keywords:** Polycyclic aromatic hydrocarbons, Carcinogenicity, BFHC indices, Fused benzene ring-based topology, QSAR

## Abstract

**Objectives:**

Predicting the carcinogenicity of polycyclic aromatic hydrocarbons (PAHs) is challenging due to their structural complexity and diverse biological activity. Carcinogenic potential is strongly influenced by molecular structure mainly ring arrangement and substitution patterns that affect metabolic activation and DNA reactivity. To improve predictive modeling, we compiled a dataset of 302 polycyclic aromatic hydrocarbons featuring novel topological indices-Bay, Fjord, Harbor, and Canyon (BFHC)-along with experimental carcinogenicity classifications, log P, and log Iball values. PAHs were further classified according to their fused-benzene ring topology as linear, angular (cata-condensed), or peri-condensed (clustered) structures. The BFHC indices were developed as part of a PhD dissertation (https://rcin.org.pl/publication/63491).

**Data description:**

The dataset provides structural and experimental information for 302 PAHs, including both unsubstituted and hydrocarbon-substituted molecules (such as alkyl, benzyl, and small ring substituents). Each compound is characterized by XYZ Gaussian input files, IUPAC name, molecular structure, carbon and hydrogen counts, and counts of four topological indices - Bay, Fjord, Harbor, and Canyon -collectively referred to as BFHC indices. The dataset also includes experimental carcinogenicity classified into seven qualitative categories, physicochemical properties such as log P (131 compounds) and log Iball (117 compounds), and fused benzene ring-based topology classification (linear, angular, peri-condensed). The dataset is publicly available and suitable for Quantitative Structure-Activity Relationships (QSAR) and machine learning applications in toxicology and carcinogenic potency of PAHs.

**Supplementary Information:**

The online version contains supplementary material available at 10.1186/s13104-026-07791-w.

## Objectives

Polycyclic aromatic hydrocarbons (PAHs) are abundant environmental pollutants that are composed of chemically diverse compounds with varying carcinogenic potential [1, 2]. Accurately predicting their carcinogenicity is challenging due to their structural diversity and the wide range of biological activities they exhibit. While many QSAR models have been proposed [3, 4], their generalizability is often limited by insufficient structural characterization or inconsistent data [5–7].

To address these gaps, we compiled a dataset of 302 PAHs, incorporating both unsubstituted and hydrocarbon-substituted molecules (e.g., alkyl, benzyl, and small ring substituents). While hydrocarbon substituents can influence local steric accessibility and metabolic processing of specific ring sites [6, 8], the present dataset does not directly encode electronic effects of substituents such as changes in electron density or HOMO–LUMO energies as such properties require quantum chemical descriptors which is outside the scope this data note.

Previous studies on substituted PAHs highlight how electron‑donating and electron‑withdrawing groups can influence metabolic activation and carcinogenic potential. Reviews of methylated and non‑methylated PAHs show that substitutions at reactive positions alter metabolic bioactivation and carcinogenic outcomes and that alkyl substituents can shift the metabolic pathways relevant to mutagenic and carcinogenic intermediates [9, 10]. Additionally, substituted derivatives such as oxygenated and nitrated PAHs often exhibit enhanced mutagenic or carcinogenic effects compared to parent PAHs [11]. These findings, combined with established evidence that several PAHs are carcinogenic in animals and humans [12]. This suggests that our current data sets could provide a good foundation for future extensions incorporating broader substituent chemistry to refine quantitative structure‑activity relationships.

We introduced novel structural indices-Bay, Fjord, Harbor, and Canyon regions (BFHC) - to characterize molecular topology along with their core fused benzene ring topology classification (linear, angular, peri-condensed) [6, 8, 13] that can be extensively used in carcinogenicity and toxicological researches of PAHs. Each PAH’s carcinogenic potency is classified based on the highest experimentally observed activity reported in peer-reviewed studies [1, 7, 14–23] providing a consistent reference scale for model development.

Hence, our focus is on BFHC indices and core fused benzene ring topology and structural concavities that have been mechanistically linked to metabolic activation pathways relevant to carcinogenic outcomes.

By integrating detailed topological descriptors (BFHC indices and along with the fused ring classes) with curated experimental carcinogenicity data and relevant physicochemical parameters, this dataset is designed to support the development and validation of improved QSAR and machine learning models for predicting PAH carcinogenicity as well as other toxicological investigations involving PAHs.

## Data description

This dataset contains structural and experimental information for 302 polycyclic aromatic hydrocarbons, all composed exclusively of carbon and hydrogen atoms. Each compound is represented by an XYZ Gaussian input file. The metadata table (PAH_Topological_Regions_Carcinogenicity_Data.xlsx in the dataset) includes the PAH xyz input file, the IUPAC name of the compound, its chemical structure. It also lists the number of carbon and hydrogen atoms, and provides BFHC indices and fused benzene ring-based topology classifications.

The BFHC indices are topological descriptors that capture the local geometry of PAH ring systems with the presence of four key structural motifs: Bay (B), Fjord (F), Harbor (H), and Canyon (C) regions. A Bay region (B) is a concave pocket formed between adjacent aromatic rings and is identified by a short four-atom path: X–sp²C–sp²C–Y, where X and Y are typically hydrogen-bearing sp² carbons [13]. Fjord regions (F) are V-shaped motifs involving a five-atom path (X–sp²C–sp²C–sp²C–Y), often introducing steric strain due to inward-facing hydrogens. Harbor regions (H) extend this pattern to six atoms (X–sp²C–sp²C–sp²C–sp²C–Y), forming wider angular cavities. Canyon regions (C) represent the deepest structural pockets, following a seven-atom sequence (X–sp²C–sp²C–sp²C–sp²C–sp²C–Y), commonly found in large or contorted PAHs. These indices provide a rich and detailed characterization of molecular topology and are especially useful for distinguishing structural isomers and understanding ring fusion patterns that may influence PAH reactivity or biological activity [18, 24].

To generate these indices, a python code was developed that uses a xyz Cartesian coordinates of the PAHs. The algorithm first calculates a distance matrix to identify bonded atoms based on covalent radii thresholds for carbon and hydrogen. An adjacency matrix is then constructed to represent molecular connectivity. Atom types and hybridizations are assigned by evaluating the degree of each atom in the adjacency matrix, distinguishing between hydrogen (H), sp, sp², and sp³ types, including hydrogen-bonded variants (e.g., sp²-H). Ring structures are identified using graph-theoretic approaches, allowing for classification by ring size (e.g., 5-, 6-, or 7-membered cycles) [25].

Topological motifs such as Bay, Fjord, Harbor, and Canyon regions are identified by detecting specific atomic paths across fused aromatic systems. For example, Bay regions are determined by a four-atom path that bridges adjacent rings through a common sp² carbon, while Fjord, Harbor, and Canyon motifs are identified by extending this path to five, six, and seven atoms, respectively, with geometric constraints to ensure structural validity. This systematic annotation enables consistent comparison across PAHs and supports high-quality feature generation for machine learning applications.

To further characterize core structure, each PAH is classified according to a fused benzene ring–based topology, reflecting how benzene rings are arranged and fused within the molecule [13, 26]. Three classifications are used: linear, angular (cata-condensed), and peri-condensed (clustered). A linear arrangement occurs when benzene rings are fused in a straight sequence, forming acene-type systems. Angular (cata-condensed) arrangements arise when rings are fused in bent or angular configurations, often associated with the formation of bay regions that influence chemical reactivity. Peri-condensed (clustered) arrangements occur in highly fused ring systems where multiple edges are shared between rings, generating compact structures with fjord, harbor, or canyon motifs.

These ring fusion topologies influence aromatic stabilization, steric strain, and the formation of bay and fjord regions, which are key structural features governing metabolic activation and carcinogenic potential in PAHs.

Each compound’s experimental carcinogenicity classification is recorded using a standardized seven-level scheme: non-carcinogenic (–), very low (±), low (L), low to moderate (LM), moderate (M), high to moderate (HM), and high (H) [1, 24]. In addition, physicochemical properties such as log P (octanol–water partition coefficient) [27, 28] and the log Iball index [29, 30] are included, where available log P values are reported for 131 compounds and log Iball values for 117 compounds.

## Methodology summary

The dataset was developed through a combination of computational modeling, manual annotation, and literature-based data curation. This has the following key information:

- Molecular Structure Generation: Molecular structures were prepared using Chem Draw while the xyz coordinates of the PAHs were prepared using Gaussian.
- Topological descriptors: Bay, Fjord, Harbor, and Canyon- BFHC indices.
- Fused benzene ring-based classification: Linear, angular (cata-condensed, peri-condensed(clustered).
- Experimental Carcinogenicity: These information was compiled from peer-reviewed published sources and harmonized into a unified, qualitative classification system with following level catagories: “-“ or “L” : non-carcinogenic; “±”: very low level (almost inactive); “+”, LM : low moderate; M: moderate; HM: high moderate; H: high potency (extremely active).
- Physicochemical Property: Log P values (octanol–water partition coefficients) and the log *Iball index* were collected from peer reviewed published literatures.
- Python Code for BFHC Indices: A Python code/algorithm was developed to automate the identification of BFHC regions using xyz atomic coordinates of PAHs.

All the aforementioned datasets are publicly available in an open-access data repository, with corresponding DOIs listed in Table 1.

**Table 1:** Overview of datasets and data files in the repository

Label	Name of data file/dataset	File type	Data repository and identifier (DOI)
Data Set 1	PAH_Topological_Regions_Carcinogenicity_Data	.xlsx	10.6084/m9.figshare.29693360.v1
Data Set 2	Pah_xyz.tar	.tar	10.6084/m9.figshare.29692925.v1
Data Set 3	Pah_Topological_Indices.py	.py	10.6084/m9.figshare.29901779.v1

## Limitations

Carcinogenicity classifications are based on the highest reported experimental effect and may reflect route or organ-specific activity rather than systemic toxicity. Physicochemical data are not fully complete, with log P reported for 131 compounds and log Iball for 117 compounds. Although the dataset includes unsubstituted and hydrocarbon-substituted PAHs (e.g., alkyl, benzyl, and small ring substituents), it does not include electronic substituent effects or heteroatom-containing functional groups.

## Supplementary Information

Below is the link to the electronic supplementary material.

Supplementary Material 1.

Supplementary Material 2.

Supplementary Material 3.

## Data Availability

The datasets described in this data note are fully publicly available and open-access in the Figshare repository at the following DOIs:- [10.6084/m9.figshare.29693360.v1](PAH_Topological_Regions_Carcinogenicity_Data.xlsx-Topological regions and carcinogenic activities)- [10.6084/m9.figshare.29692925.v1] (Pah_xyz.tar-PAHs structure archive) and [10.6084/m9.figshare.29901779.v1] (Pah_Topological_Indices.py-Python script for producing BFHC indices).

## Abbreviations

BFHC: Bay, Fjord, Harbor, Canyon indices
DNA: Deoxyribonucleic Acid
HOMO: Highest Occupied Molecular Orbital
IUPAC: International Union of Pure and Applied Chemistry
LUMO: Lowest Unoccupied Molecular Orbital
PAH(s): Polycyclic Aromatic Hydrocarbon(s)
QSAR: Quantitative Structure–Activity Relationship
log Iball: Iball Index descriptor for carcinogenic activity (logarithmic)
log-P: Octanol–water partition coefficient (logarithmic)
